# Identifying celiac disease-related chemicals by transcriptome-wide association study and chemical-gene interaction analyses

**DOI:** 10.3389/fgene.2022.990483

**Published:** 2022-09-02

**Authors:** Mengnan Lu, Ruoyang Feng, Yuesheng Liu, Yujie Qin, Hongyang Deng, Yanfeng Xiao, Chunyan Yin

**Affiliations:** ^1^ Department of Pediatrics, The Second Affiliated Hospital of Xi’an Jiao Tong University, Xi’an, China; ^2^ Department of Joint Surgery, HongHui Hospital, Xi’an Jiao Tong University, Xi’an, China

**Keywords:** immune-mediated diseases, celiac disease, GWAS, TWAS, CGSEA

## Abstract

Celiac disease (CeD) is one of the most common intestinal inflammatory diseases, and its incidence and prevalence have increased over time. CeD affects multiple organs and systems in the body, and environmental factors play a key role in its complex pathogenesis. Although gluten exposure is known to be the causative agent, many unknown environmental factors can trigger or exacerbate CeD. In this study, we investigated the influence of genetic and environmental factors on CeD. Data from a CeD genome-wide association study that included 12,041 CeD cases and 12,228 controls were used to conduct a transcriptome-wide association study (TWAS) using FUSION software. Gene expression reference data were obtained for the small intestine, whole blood, peripheral blood, and lymphocytes. We performed Gene Ontology and Kyoto Encyclopedia of Genes and Genomes enrichment analyses using the significant genes identified by the TWAS and conducted a protein–protein interaction network analysis based on the STRING database to detect the function of TWAS-identified genes for CeD. We also performed a chemical-related gene set enrichment analysis (CGSEA) using the TWAS-identified genes to test the relationships between chemicals and CeD. The TWAS identified 8,692 genes, including 101 significant genes (*p*_*adjusted*_ < 0.05). The CGSEA identified 2,559 chemicals, including 178 chemicals that were significantly correlated with CeD. This study performed a TWAS (for genetic factors) and CGSEA (for environmental factors) and identified several CeD-associated genes and chemicals. The findings expand our understanding of the genetic and environmental factors related to immune-mediated diseases.

## Introduction

Celiac disease (CeD) is one of the most common intestinal inflammatory diseases, and it is characterized by small intestine inflammation, villous atrophy, crypt hyperplasia, and malabsorption (Kahaly et al., 2018). CeD is present worldwide, and its prevalence varies by continent, with cases occurring in Northern and Western Europe, South America (1.3%), and Asia (1.8%) (Lebwohl and Rubio-Tapia, 2021). In addition, the incidence and prevalence of CeD have increased over time (King et al., 2020). The key factors underlying the pathogenesis of CeD include environmental triggers (gluten, olmesartan, gut bacteria, etc.), genetic predisposition (HLA-DQ2 or HLA-DQ8), autoantigens (TG2), adaptive immune response activation (CD4^+^ T and B cells), and gluten-induced alterations in the intestinal epithelium after intraepithelial cytotoxic lymphocyte activation (Verdu and Schuppan, 2021). The clinical presentation of CeD is divided into intestinal and extraintestinal manifestations. The intestinal form of CeD is more commonly detected in pediatric patients and is characterized by diarrhea, loss of appetite, and growth limitation (Caio et al., 2019). With the development of diagnostic technology, novel features of CeD are being revealed. CeD affects multiple organs and systems throughout the body, including the skin (dermatitis), musculoskeletal joints (myositis and arthritis), blood (anemia), spleen, endocrine glands, lungs, and heart, and it can lead to gynecological (infertility and abortion), neurological, and psychiatric problems, as well as malignancy (lymphoma and adenocarcinoma). CeD can be successfully treated with a gluten-free diet (GFD); however, this treatment strategy may considerably affect the quality of life (Vriezinga et al., 2015). Thus, biomarkers must be identified to determine the risk factors and develop potential interventions for high-risk groups (Auricchio and Troncone, 2021).

In recent years, the most common single nucleotide polymorphisms (SNPs) have been assessed in genome-wide association studies (GWASs) to identify statistical associations with various complex traits (Frazer et al., 2009). The SNPs identified through GWASs may provide strongly predictive and prognostic information or identify important pharmacological implications (Manolio, 2013). Therefore, GWASs could lead to a better understanding of diseases and treatments (Hirschhorn and Daly, 2005). GWASs have been used to reveal the polygenetic basis of common diseases, especially autoimmune diseases (Inshaw et al., 2018), such as multiple sclerosis, inflammatory bowel disease (Yang et al., 2021), systemic lupus erythematosus (Lu et al., 2021), and rheumatoid arthritis (Ha et al., 2021).

However, the reliability of GWASs for assessing the risk of complex diseases is limited because most SNPs identified by GWASs are located in noncoding regions of the disease genome (Xu et al., 2021). Genetic loci cause variations in human traits, including growth, fitness, and disease; therefore, studies on the genetics of gene expression have emerged as a key tool for linking DNA sequence variations to phenotypes (Albert and Kruglyak, 2015). Transcriptome-wide association studies (TWASs) represent an effective method of identifying significant expression-trait associations, and this method substantially outperforms its cis-expression quantitative trait locus (eQTL) analog, both in imputing the expression and associations with a trait (Gusev et al., 2016). A recent study performed a TWAS for inflammatory bowel disease (IBD) and identified 78 novel susceptibility genes associated with IBD (Díez-Obrero et al., 2022). Gastrointestinal autoimmune disorders, including CeD, IBD, autoimmune pancreatitis, and autoimmune liver disease, are caused by the complex interplay between genetic and environmental factors (Rossi et al., 2022). Therefore, TWAS is a good method for investigating gene expression in different tissues.

**FIGURE 1:**
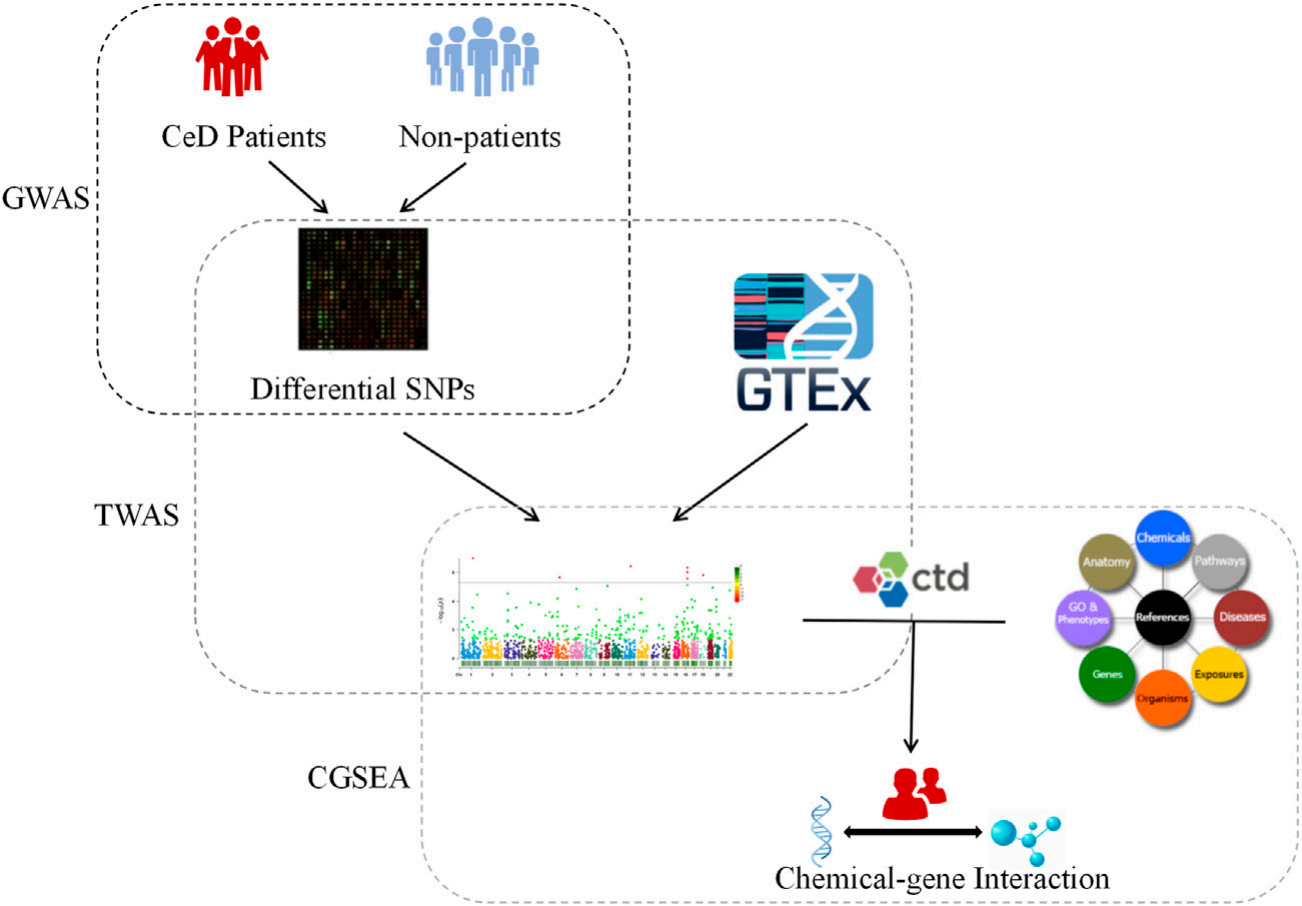
Flow chart. CeD: Celiac disease; GWAS: Genome-wide association studies; TWAS: Transcriptome-wide association studies; GTEx: Genotype-Tissue Expression Project Database; CTD: Comparative Toxicogenomics Database; CGSEA: Chemical-related gene set enrichment analysis.

The present study aimed to investigate the influence of genetic factors on CeD by performing a TWAS based on a GWAS dataset that includes gene expression data for the small intestine, whole blood, peripheral blood, and lymphocytes. We also reevaluated the expression of genes identified by the TWAS, performed a gene function analysis, and identified CeD-associated chemicals. This study expands our understanding of the genetic and environmental factors affecting CeD (Figure 1).

## Methods

### CeD GWAS summary data

We used published GWAS summary data for CeD (Trynka et al., 2011). The analyzed data included 12,041 celiac disease cases and 12,228 controls, and the data were from 7 countries, including the UK (NCeliac cases = 7,728, NControls = 8,274), the Netherlands (NCeliac cases = 1,123, NControls = 1,147), Poland (NCeliac cases = 505, NControls = 533), Spanish Consortium for the Genetics of Celiac Disease (NCeliac cases = 545, NControls = 308), Spain (Madrid) (NCeliac cases = 537, NControls = 320), Italy (Rome, Milan, and Naples) (NCeliac cases = 1,374, NControls = 1,255), and India (Punjab) (NCeliac cases = 229, NControls = 391). This study included large resequencing sample sizes from cases and controls after stringent data quality control as indicated in the Online Methods (ncbi.nlm.nih.gov/pmc/articles/PMC3242065/#SD5). Dense genotyping strategy and stepwise conditional association analyses have been performed to identify the complex architecture of multiple common and rare genetic risk variants. Although Gosia Trynka et al. localized signals at many loci, more detailed functional studies are required to demonstrate which gene variants might be causal.

### TWAS of CeD

TWAS is a powerful method that integrates gene expression with GWAS to identify genes that are associated with certain traits. The TWAS approach is better than a linkage disequilibrium-based (LD-based) estimate of local genetic correlation; therefore, it is appropriate for the study of the genetic etiology of multiple phenotypes (Gusev et al., 2016). To measure significant SNP-trait associations, all genome-wide testing burdens have been corrected to ensure that the TWAS false positive rate is well-controlled. The software program FUSION (default settings) was used for the TWAS and joint analyses of regions containing multiple significant associations (Pain et al., 2019). The most popular TWAS methods, such as PrediXcan, TWAS-Fusion, and SMR, test causal relationships between gene-expression levels and complex traits (Zhang et al., 2020), among which, the TWAS-Fusion method is used more often. Briefly, Bayesian sparse linear-mixed models (Zhou et al., 2013) were used to calculate SNP expression weights for specific genes at the 1-Mb cis position and estimate the association of predicted expression levels with CeD using the following formula: Ztwas = w + Z/(w × [Lw]1/2) (Gusev et al., 2016), where w denotes the weight, Z denotes the Z-score, and L denotes the SNP correlation matrix (definition, LD). Each feature expanded in 100,000 bp was defined contiguous. The Minium *p*-value to include feature in the joint model was 0.05. Features with r^2^ greater than 0.9 would be considered identical. And Features with r^2^ less than 0.008 would be considered independent. The diagnosis of CeD relies on serological tests, small bowel endoscopy, and pathological biopsy. Thus, we used the gene expression weights for the small intestine, whole blood, peripheral blood, and lymphocytes as references, and they can be downloaded from the FUSION website (http://gusevlab.org/projects/fusion/). All *p* values are then subjected to multiple testing correction using the Benjamini-Hochberg procedure to gather Q values, which represent the minimum false discovery rate (FDR) threshold at which the contact is deemed significant.

### TWAS-based functional exploration analysis

We constructed a Venn plot to identify the common and tissue-specific genes that were expressed among the small intestine, whole blood, peripheral blood, and lymphocytes. The Kyoto Encyclopedia of Genes and Genomes (KEGG) (Kanehisa and Goto, 2000) and Gene Ontology (GO) (Hill et al., 2002) enrichment analyses were performed to identify and confirm related biological processes. The Venn plot and KEGG and GO enrichment were performed using the R packages “ggplot2,” “org.Hs.eg.db,” and “clusterProfiler” (R Foundation for Statistical Computing, Vienna, Austria. https://www.R-project.org/). We generated a protein–protein interaction (PPI) network using the STRING v11.5 database (STRING, https://string-db.org), which required a confidence score of 0.15 and “active interaction sources,” based on a previous study (Jensen et al., 2009). We used Cytoscape to visualize all the interaction networks (Shannon et al., 2003) and the plugin Molecular Complex Detection (MCODE) for the module analysis (Bader and Hogue, 2003).

### Gene expression profiles of CeD

We downloaded gene profiles (GSE72625) from the Gene Expression Omnibus (GEO) database (https://www.ncbi.nlm.nih.gov/geo/). This study examined the gene expression profile in pars descendens of duodenum in celiac disease patients (*n* = 10, Marsh grade 3a or above) and healthy controls (*n* = 17) by gene expression microarray. We further analyzed the differential gene expression of small intestinal genes, and details on the samples can be found in the original article (Jørgensen et al., 2016). GSE72625 was downloaded from the GEO database through the GEOquery package. If probes corresponding to multiple molecules were removed, and if probes corresponding to the same molecule were encountered, only the probe with the largest signal value was retained. Statistical analysis and visualization were performed using the R packages “GEOquery” (Davis and Meltzer, 2007), “limma” (Smyth and Gentleman, 2005), “ComplexHeatmap” (Gu et al., 2016), and “ggplot2.” Differentially expressed genes (DEGs) were identified based on |log2FC|>1 and adjusted *p*-values<0.05. Further analyses for DEGs were performed using the R packages “org.Hs.eg.db” and “clusterProfiler.”

### Chemical-related gene set enrichment analysis

The chemical gene expression annotation dataset used in this study was downloaded from the Comparative Toxicology Genomics Database (CTD) (http://ctdbase.org/downloads/). The CTD provides four datasets, namely, chemical gene interaction function, chemical disease association, genetic disease association, and chemical element phenotypic association, and it integrates the four datasets to automatically construct a hypothetical chemical gene phenotypic disease network to illustrate the molecular mechanisms underlying diseases that are affected by the environment (Mattingly et al., 2004). Cheng et al. downloaded and used 1,788,149 chemical-gene pair annotation terms driven by humans and mice and generated 11,190 chemical substance-related gene sets (Cheng et al., 2020a). The CGSEA is a flexible tool for assessing associations between chemicals and complex diseases, and the detailed analysis method is provided in the original article (Cheng et al., 2020a). In the present study, we performed 10,000 permutations to obtain the empirical distribution of the GSEA statistical data (Mooney and Wilmot, 2015) for each chemical, and then calculated the *p*-value of each chemical based on the empirical distribution of the CGSEA data. Based on previous studies (Cheng et al., 2020b), we excluded gene sets containing less than 10 or more than 200 genes to control for the influence of gene set size on the results.

## Results

### TWAS of CeD

**FIGURE 2:**
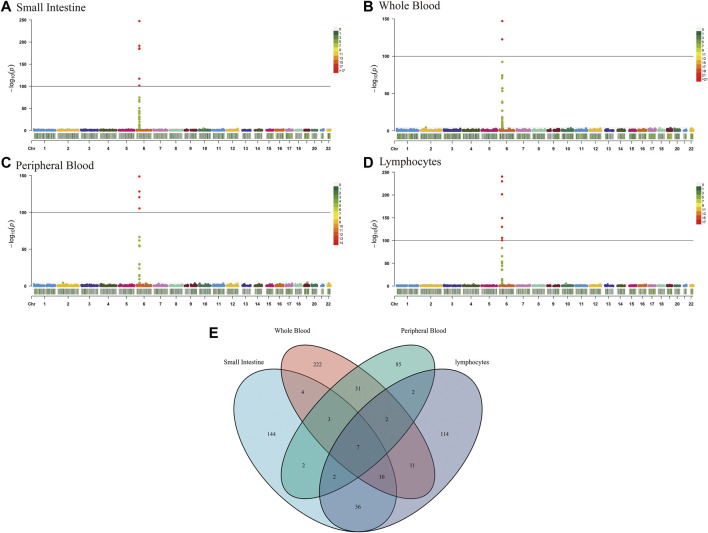
Manhattan plots of association results from the CeD transcriptome-wide association study and functional exploration of seven novel TWAS-identified CeD-susceptibility genes. Each dot represents the genetically predicted expression of one specific gene for the small intestine, whole blood, peripheral blood, and lymphocytes tissues prediction models. The x axis represents the genomic position of the corresponding gene, and the y axis represents the negative logarithm of the association *p*-value. **(A)** Gene expression weights for the small intestine. **(B)** Gene expression weights for whole blood. **(C)** Gene expression weights for peripheral blood. **(D)** Gene expression weights for lymphocytes. **(E)** Venn diagram reveals the overlap of TWAS-identified genes in different tissues. Blue, small intestine; red, whole blood; green, peripheral blood; purple, lymphocytes.

The TWAS identified a total of 675 unduplicated genes were identified (*P*_*TWAS*_ < 0.05, MODELCV. R^2^ ≥ 0.01; Figure 2), including 208, 289, 134, and 184 genes for the small intestine, whole blood, peripheral blood, and lymphocytes, respectively (Supplementary Table S1).

Tissues have unique gene expression profiles. Thus, we performed an overlap analysis of the 675 TWAS-identified genes in different tissues to identify the representatively expressed genes and commonly expressed genes. Figure 1E illustrates the resulting Venn diagram, which indicates the number of genes expressed in one or more tissues. Seven significant TWAS-identified commonly expressed genes were associated with CeD in the small intestine, whole blood, peripheral blood, and lymphocytes. These 7 CeD-susceptibility genes identified by TWAS were *TCF19* (Transcription Factor 19; chromosome 6), *HLA-DQA1* (major Histocompatibility Complex, class II, DQ alpha 1; chromosome 6), *MICB* (MHC class I Polypeptide-related sequence B; chromosome 6), *AP3S2* (Adaptor-related protein complex 3 Subunit sigma 2; chromosome 15), *HEATR3* (HEAT Repeat Containing 3; chromosome 16), *GSDMB* (Gasdermin B; chromosome 17), and *POLI* (DNA Polymerase Iota; chromosome 18). Table 1 presents detailed information on the 7 genes, including the rsID of the most significant GWAS SNPs at the locus (i.e., BEST. GWAS.ID) and the TWAS *p*-values (i.e., *p* TWAS).

**TABLE 1:** TWAS-identified expressed CeD-susceptibility genes in four tissues.

Gene	BEST.GWAS.ID	*p*_*TWAS*_			
		Small intestine	Whole blood	Peripheral blood	Lymphocytes
*TCF19*	rs3130923	9.07E-04	1.05E-16	5.50E-10	1.90E-02
*HLA-DQA1*	rs2854275	5.77E-04	3.40E-93	2.63E-67	3.06E-06
*MICB*	rs497309	5.08E-29	2.81E-10	2.35E-02	5.30E-15
*AP3S2*	rs6496609	3.85E-02	4.46E-02	9.39E-03	2.58E-02
*HEATR3*	rs6500249	3.45E-02	1.69E-02	2.03E-03	2.71E-02
*GSDMB*	rs9916158	2.38E-02	2.99E-02	3.87E-02	2.63E-02
*POLI*	rs508218	1.59E-02	1.39E-02	8.23E-03	2.70E-02

### Functional exploration of TWAS-identified significant CeD-susceptibility genes

**FIGURE 3:**
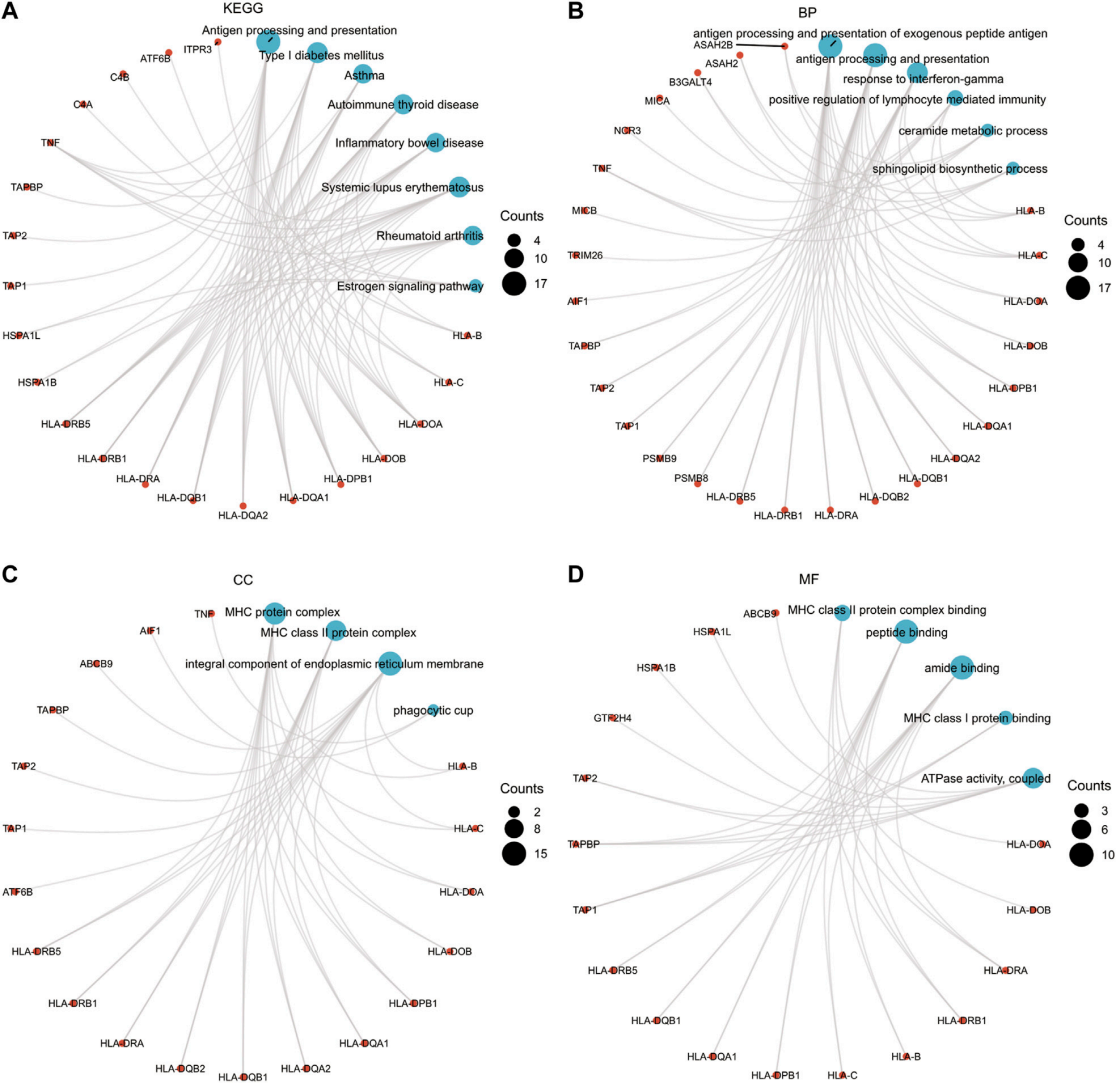
Functional exploration of TWAS-identified significant CeD-susceptibility genes. **(A)** Network diagrams of Kyoto Encyclopedia of Genes and Genomes functional analysis; **(B)** Network diagrams of Biological Process functional analysis; **(C)** Network diagrams of Cellular Component functional analysis; **(D)** Network diagrams of Molecular Function functional analysis.

97 TWAS-identified significant CeD-susceptibility genes among four tissues were identified by FDR multiple comparison correction (*P*_*FDR*_ < 0.05, MODELCV. R2 ≥ 0.01; Supplementary Table S2). We subjected the 101 TWAS-identified significant CeD-susceptibility genes to molecular function studies based on KEGG and GO analyses (Figure 3). There were eight KEGG categories including Antigen processing and presentation, Type I diabetes mellitus, Asthma, Autoimmune thyroid disease, Inflammatory bowel disease, Systemic lupus erythematosus, Rheumatoid arthritis, and Estrogen signaling pathway. Six enriched GO terms belonged to the biological process category, including antigen processing and presentation of exogenous peptide antigen, antigen processing and presentation, response to interferon-gamma, positive regulation of lymphocyte mediated immunity, ceramide metabolic process, and sphingolipid biosynthetic process. Four significantly enriched GO terms belonged to the cellular component category, including MHC protein complex, MHC class II protein complex, integral component of endoplasmic reticulum membrane, and phagocytic cup. In terms of the molecular function category, the enriched GO terms involved MHC class II protein complex binding, MHC class I protein binding, ATPase activity, peptide binding, and amide binding.

### PPI network of the TWAS-identified significant genes

**FIGURE 4:**
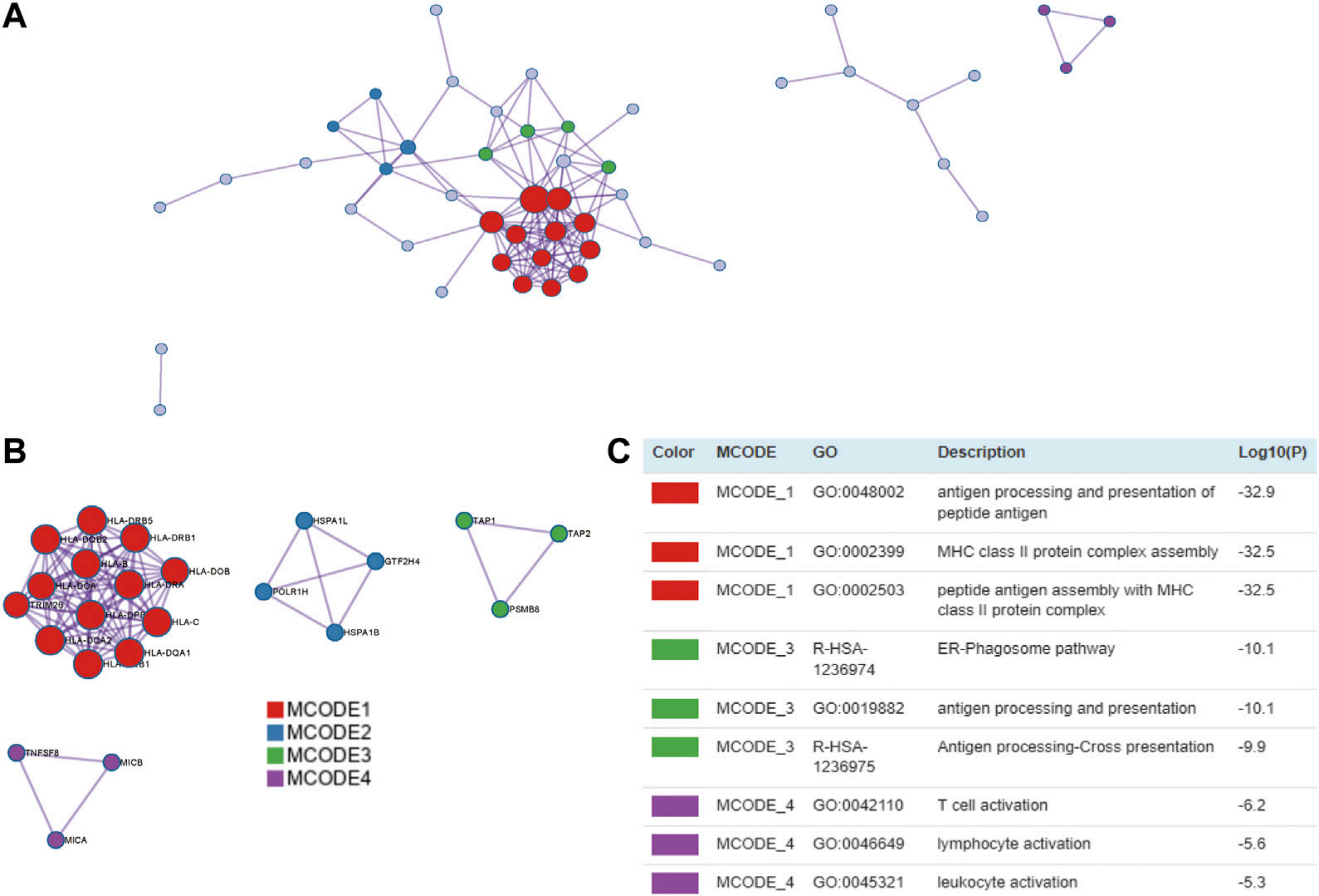
PPI network and significant modules. Red and blue circles indicate upregulated and downregulated TWAS-identified genes. **(A)** PPI network of the TWAS-identified significant genes. **(B)** Significant Molecular Complex Detection (MCODE) algorithm of the PPI network. **(C)** Functional exploration of MCODE.

We used 97 TWAS-identified significant CeD-susceptibility genes for a PPI analysis and successfully transformed 87 protein-coding genes (Figure 4A). To effectively identify densely connected regions of the PPI network, we formed six MCODE clusters with PPI network genes (Figure 4B). The hub genes identified by the MCODE plugin were further analyzed for functional exploration (Figure 4C). MCODE1 was characterized by MHC class II protein complex. MCODE3 were related to ER-Phagosome pathway and antigen processing. MCODE4 associated with leukocyte activation.

### Common genes shared by TWAS and mRNA expression profiling

**FIGURE 5:**
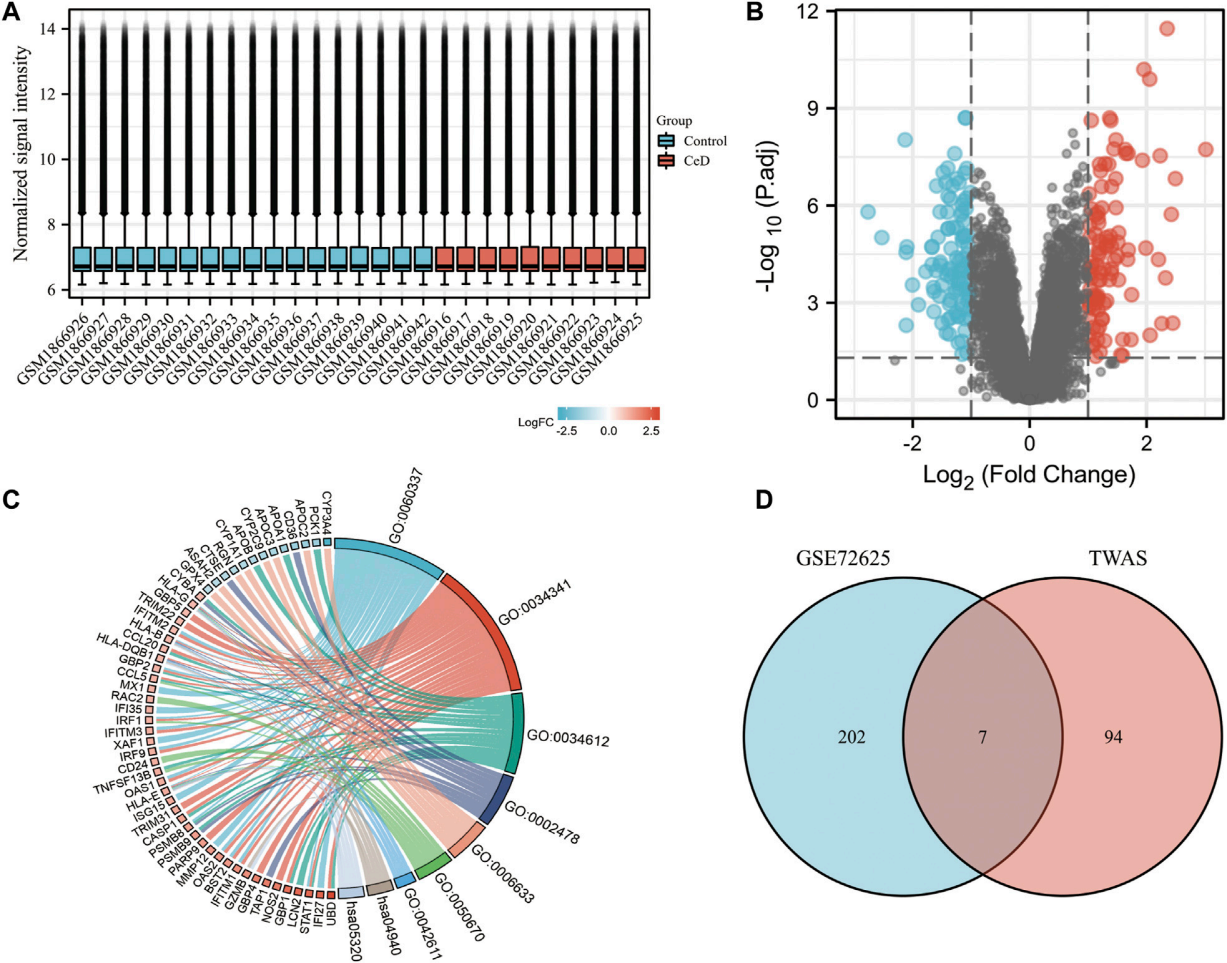
Gene expression profiles of CeD. **(A)** Normalized bar plot of the GSE72625 dataset. **(B)** Vocanol plot of the GSE72625 dataset. Gene expression analysis of the GSE113469 dataset. **(C)** Circle diagrams of Kyoto Encyclopedia of Genes and Genomes functional analysis. **(D)** Venn diagram reveals the overlap of differentially expressed genes of GSE72625 and TWAS-identified genes.

To verify the reliability of the TWAS-identified significant CeD-susceptibility genes, we selected and analyzed GEO dataset (GSE72625). GSE72625 dataset were normalized and corrected (Figure 5A). GSE72625 (Figure 5B) contained 209, respectively, and an enrichment analysis suggested that the DEGs were associated with immune-related pathways, such as the MHC protein complex, response to tumor necrosis factor, response to interferon-gamma, and regulation of lymphocyte proliferation (Figure 5C).

We compared the genes identified by the TWAS with the DEGs identified in the two selected datasets. We detected 7 common genes among the DEGs from the TWAS and GSE72625 (Figure 5D). The common genes are listed in Table 2.

**TABLE 2:** Common genes identified by TWAS with GSE 72625 mRNA expression profiles.

Gene	*p*_*adjusted*_	log2FC	Chromosome	BEST.GWAS.ID
*ASAH2*	1.04E-04	-1.20	10	rs10821669
*HCP5*	1.20E-11	1.65	6	rs497309
*HLA-B*	1.63E-06	1.03	6	rs3115672
*HLA-DQB1*	5.80E-03	1.09	6	rs2854275
*PSMB8*	3.05E-13	1.37	6	rs2854275
*PSMB9*	4.72E-05	1.45	6	rs2854275
*TAP1*	6.32E-11	1.95	6	rs2854275

### CGSEA of the TWAS-identified genes

**FIGURE 6:**
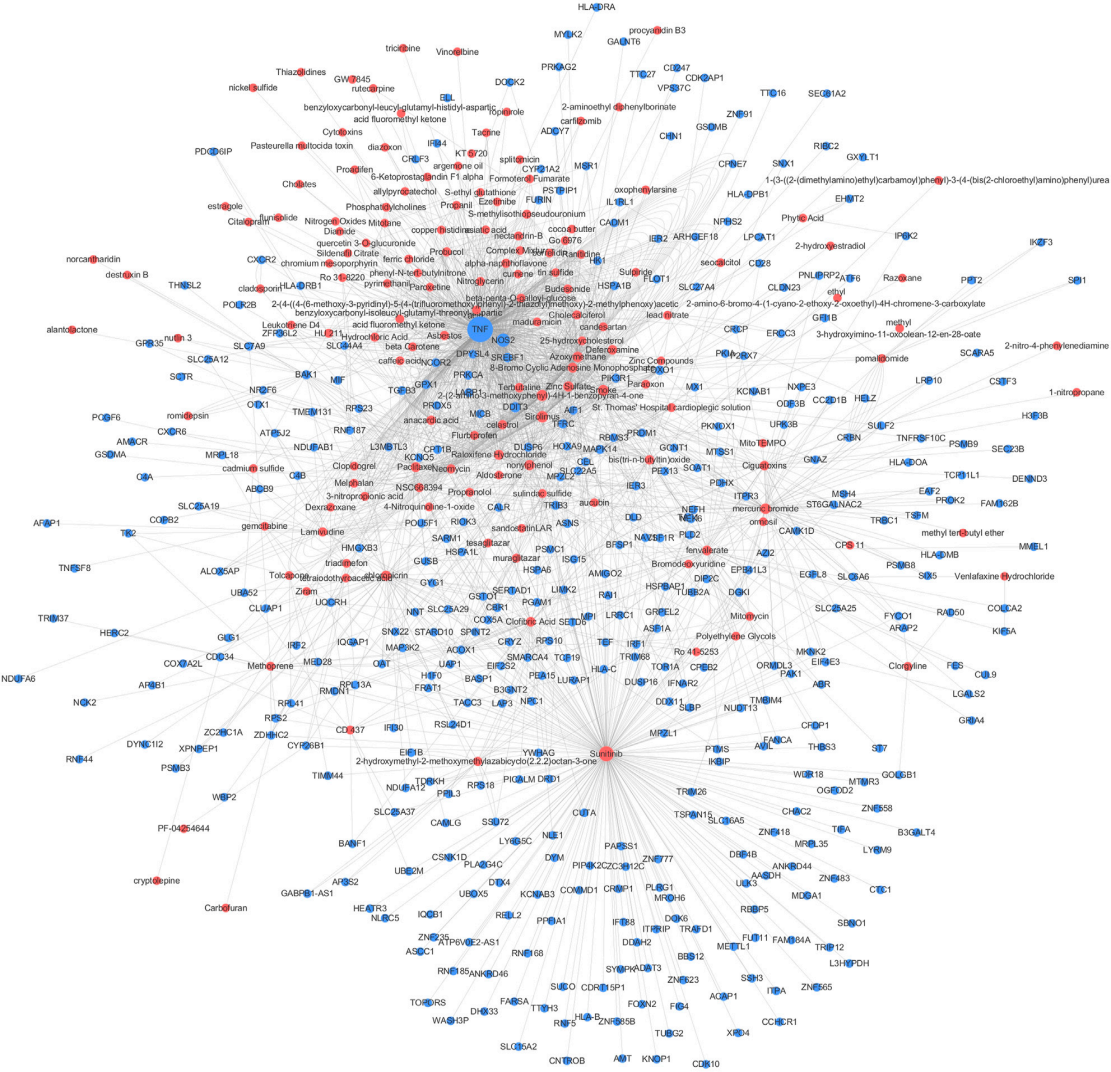
CGSEA analysis results. Network of chemicals and their target genes based on the TWAS-identified genes. Red and blue circles indicate chemicals (*p*_*CFSEA*_ < 0.05) and TWAS-identified genes, respectively. The size of the circle indicates the value degree with other points.

We performed a CGSEA to investigate the environmental factors influencing CeD, and it revealed 2,559 chemicals, including 178 chemicals correlated with CeD (Supplementary Table S3). Our constructed network of chemicals and their target genes based on the TWAS-identified genes is illustrated in Figure 6. The absolute value of the normalized enrichment score (NES) > 1 is considered significantly enriched according to the GSEA, and 25 significantly enriched chemicals were identified, with |NES|>1 and *p*-value<0.05 (Table 3).

**TABLE 3:** Significantly enriched chemicals identified by the CGSEA for TWAS-identified significantly expressed genes associated with CeD.

Chemicals ID	Chemical name	*p*_*CFSEA*_	NES
D005996	Nitroglycerin	3.00E-04	34.45
C045934	4-Hydroxy-2-hexenal	4.00E-03	3.98
D053778	Thiazolidines	3.60E-03	3.97
C042720	Mercuric bromide	1.22E-02	3.32
D010713	Phosphatidylcholines	6.80E-03	3.30
D002995	Clofibric Acid	1.76E-02	3.00
C100187	Chloropicrin	2.21E-02	2.61
D017255	Acitretin	8.40E-03	2.19
C500085	Muraglitazar	8.50E-03	2.08
C004363	Alantolactone	7.30E-03	1.98
D002922	Ciguatoxins	2.62E-02	1.92
D002235	Carbofuran	2.30E-03	1.83
C017558	Nickel sulfide	3.20E-03	1.71
D012906	Smoke	5.00E-02	1.54
C459559	Vaticanol C	5.00E-03	1.52
D020123	Sirolimus	9.60E-03	1.43
C098468	Copper histidine	7.50E-03	1.34
D019287	Zinc sulfate	4.02E-02	1.32
D002996	Clomiphene	1.19E-02	1.28
C007734	Flunisolide	4.90E-03	1.27
D045424	Complex mixtures	3.80E-03	1.23
D020355	Cholates	7.00E-04	1.20
C087123	Romidepsin	1.82E-02	1.19
D000077235	Vinorelbine	1.22E-02	1.15
C040424	Destruxin B	1.15E-02	1.02

## Discussion

CeD occurs in approximately 1% of people in most populations globally, and the true incidence rate is rising (Lebwohl et al., 2018). CeD is a multisystem disorder that commonly affects the digestive system, although it can also affect the dermatologic, hematologic, neurologic, musculoskeletal, endocrine, and reproductive systems (Rubin and Crowe, 2020). CeD is diagnosed based on serological tests and gastrointestinal biopsies; therefore, studying changes in gene expression in the digestive tract and blood can help provide new information for identifying biomarkers and understanding the etiology of CeD.

We performed a comprehensive TWAS to predict the relationship between CeD and significant genes found in the small intestine, whole blood, peripheral blood, and lymphocytes. Of particular interest were the seven significant TWAS-identified common genes associated with CeD found in all four tissues, which included five novel genes (*TCF19*, *AP3S2*, *HEATR3*, *GSDMB*, and *POLI*) and two genes within previously GWAS-identified CeD loci (*HLA-DQA1* (Coleman et al., 2016) and *MICB* (González et al., 2004)). *ASAH2* is a new gene associated with CeD, which identified by TWAS and mRNA expression profiles. Neurodegenerative diseases occur more frequently in patients with inflammatory gastrointestinal diseases including IBD or CeD, while *ASAH2* has been discovered in Parkinson’s disease (Blokhin et al., 2022) and Alzheimer’s disease (Avramopoulos et al., 2007). Thus, *ASAH2* might play a key role in the gut-brain axis of CeD patients. We subjected the 97 TWAS-identified significant CeD-susceptibility genes to enrichment analyses and found that they were associated with the MHC protein complex and immune processes, which is similar to the findings of a recent study (Høydahl et al., 2019). Our PPI analysis of 87 protein-coding genes also provided further support for these findings. In addition, our study also identified an association with the estrogen signaling pathway and proteins, which may partially explain the fertility problems caused by CeD and provide new directions for the treatment of CeD complications. Therefore, our study provides new information that improves our understanding of the genetics and etiology of CeD.

Environmental factors play a key role in the complex pathogenesis of CeD. Although gluten exposure is known to be a causative agent, many unknown environmental factors may trigger or exacerbate CeD (Leonard et al., 2020). We extended the classic GSEA approach to detect the association between environmental chemicals and CeD from the published GWAS summary datasets and identified 178 chemicals, including 25 significantly enriched chemicals. Patients with untreated CeD may develop cardiovascular problems, including cardiovascular risk, stroke, thrombosis, atherosclerosis, arterial function, and ischemic heart disease (Ciaccio et al., 2017). One possible reason for these findings is that endothelial dysfunction in patients with CeD is accompanied by lower flow-mediated vasodilation, which corresponds to the positive nitroglycerin-dependent dilation test in patients with CeD (Sari et al., 2012). Nitroglycerin was the most significantly enriched chemical based on the CGSEA, which further supports the theory that cardiovascular complications often occur along with CeD. 4-Hydroxy-2-hexenal is a lipid peroxide, and its content increases in a time- and temperature-dependent manner during seafood baking (Hu et al., 2022); moreover, it is an environmental factor that affects microbiota distributions (Zhang et al., 2021). Studies have shown that CeD is influenced by the intestinal microbiota (Lamas et al., 2020), and the associated GFD treatment also affects the composition of the intestinal microbiota and its metabolites (Zafeiropoulou et al., 2020). A GFD treatment requires the strict abstinence from foods containing wheat gluten and promotes the intake of vegetables, meat, nuts, and seafood. As seafood intake increases, the intake of 4-hydroxy-2-hexenal is likely to increase as well; thus, whether 4-hydroxy-2-hexenal may affect the course and treatment of CeD would be worth investigating. Our study also showed that CeD is associated with certain heavy metals, which may be related to the association between a GFD and heavy metal accumulation. A population-based, cross-sectional study showed that fish and rice products are suspected sources of heavy metals and people following a GFD had markedly higher levels of heavy metals in their urine and blood compared with the controls (Raehsler et al., 2018). In the present study, we identified a few energy metabolic pathways and lipid metabolic pathways via enrichment analyses and revealed several chemicals related to lipid metabolism, such as thiazolidines, clofibric acid, muraglitazar, sirolimus, and flunisolide. These results are in line with those of previous studies. Research suggests that a GFD may correspond to a high energy and fat load (Forchielli et al., 2015), which means that such a diet may lead to lipid and protein overload as well as fiber, iron, and calcium deficiencies (Valitutti et al., 2017). Children with CeD may have significant lipid abnormalities (Salardi et al., 2017), while adults with CeD are at an increased risk for metabolic syndrome (Tortora et al., 2015). The prevalence of CeD is higher in women than in men, and women may experience decreased fertility for up to 2 years before diagnosis (McAllister et al., 2019). A large cohort study suggested that compared to women without CD, women (aged 25–29 years) diagnosed with CD had a 40% relative increase in fertility problems, which corresponded to an absolute excess risk of 0.5% (Dhalwani et al., 2014). Clomiphene was identified by the CGSEA analysis, which suggested that this drug may be an effective agent for enhancing fertility in female patients with CeD. Cohort studies have shown that immune-mediated diseases are strongly associated with an increased risk of cancers (He et al., 2021). The nationwide cohort also suggested that patients with CeD have an increased risk of small bowel adenocarcinoma and adenomas (Emilsson et al., 2020). We found that certain chemicals associated with cancer were enriched, including alantolactone, vaticanol C, romidepsin, vinorelbine, and destruxin B. These results support the association between immune-mediated diseases and cancers. CeD is associated with several autoimmune diseases and asthma (Krishna et al., 2019), and numerous studies have shown that cigarette exposure is associated with the development of allergies and asthma (Murrison et al., 2019). Studies have also shown that cigarette smoke is a risk factor for RA (Heluany et al., 2021), IBD (van der Sloot et al., 2020), and colorectal tumors (van der Sloot et al., 2022). A meta-analysis of seven studies with 307,924 participants suggested that current smokers presented a markedly decreased risk of CeD compared with never-smokers (Wijarnpreecha et al., 2018). The relationship between smoking and CeD remains to be studied; however, our findings highlight the importance of studying the effects of smoking on CeD.

This study had several limitations. First, the pooled GWAS data are predominantly from European and Indian populations. Therefore, our results should be used with caution when studying other populations. Additionally, a few significant genes related to CeD obtained from the analysis have not been verified via molecular biology experiments, which should be performed in future studies. Further, certain chemicals identified in our study were previously demonstrated to play a role in other immune-mediated diseases, while others have not yet been validated, which will require additional clinical observations and cohort studies. However, to the best of our knowledge, this is the first large study that applied a CGSEA analysis to identify candidate chemicals related to CeD. TWAS can detect genes associated with CeD at the DNA level, and the CGSEA extended the classic GSEA approach to detect the association between environmental chemicals and CeD.

## Conclusion

This study aimed to determine the influence of genetic and environmental factors on CeD. The TWAS and CGSEA performed in this work revealed multiple CeD-associated genes and chemicals. This study expands our understanding of the genetic and environmental factors affecting CeD.

## Data Availability

The original contributions presented in the study are included in the article/Supplementary Material, further inquiries can be directed to the corresponding authors.
